# Protective effects of leonurine against ischemic stroke in mice by activating nuclear factor erythroid 2‐related factor 2 pathway

**DOI:** 10.1111/cns.13146

**Published:** 2019-05-13

**Authors:** Yan‐Zhao Xie, Xiang‐Jian Zhang, Cong Zhang, Yang Yang, Jun‐Na He, Yan‐Xia Chen

**Affiliations:** ^1^ Department of Neurology The Second Hospital of Hebei Medical University Shijiazhuang China; ^2^ Hebei Key Laboratory of Vascular Homeostasis and Hebei Collaborative Innovation Center for Cardio‐cerebrovascular Disease Shijiazhuang China; ^3^ The First Hospital of Hebei Medical University Shijiazhuang China; ^4^ Department of Endocrinology Second Hospital of Hebei Medical University Shijiazhuang China

**Keywords:** ischemic stroke, leonurine, nuclear factor erythroid 2‐related factor 2, oxidative stress, vascular endothelial growth factor

## Abstract

**Aims:**

Leonurine has been shown to trigger antioxidant responses during ischemic stroke, and nuclear factor erythroid 2‐related factor 2 (Nrf‐2) imparts protective effects against oxidative injury. The present study has determined that leonurine prevents ischemic injury of brain tissues via Nrf‐2 pathway activation.

**Methods:**

Male ICR mice and Nrf‐2^−/−^ mice were subjected to permanent middle cerebral artery occlusion (pMCAO) and received leonurine treatment at 2 hours after pMCAO by intraperitoneal injection. Neurological deficit scores as well as infarct volume were assessed to determine the neuroprotective role of leonurine. Nrf‐2 was investigated using Western blotting and real‐time polymerase chain reaction (RT‐PCR) analysis to elucidate the neuroprotective mechanism of leonurine. Commercial kits were employed to determine reactive oxygen species (ROS), superoxide (SOD), catalase (CAT), glutathione peroxidase (GSH‐Px), malonaldehyde (MDA), and glutathione (GSH). Vascular endothelial growth factor (VEGF) was evaluated by Western blotting and RT‐PCR analysis, and VEGF was localized using immunofluorescence.

**Results:**

The application of leonurine on ICR mice resulted in an improvement in neurological deficit scores and a reduction in infarct volume. Leonurine upregulated nuclear Nrf‐2 protein and increased total Nrf‐2 protein expression and mRNA levels. Leonurine regulated SOD, MDA, CAT, GSH, and GSH‐Px, and it significantly inhibited ROS production in ICR mice. Leonurine improved VEGF expression and increased VEGF expression in neurons, astrocytes, and endothelial cells. However, leonurine had no obvious beneficial effects on Nrf‐2^−/−^ mice.

**Conclusions:**

Leonurine exerted neuroprotective effects, promoted antioxidant responses, and upregulated VEGF expression by activating the Nrf‐2 pathway.

## 1 INTRODUCTION

Leonurine (C14H21N3O5) is one of the effective components of Herba leonuri. Prior evaluations have shown that leonurine exerted neuroprotective effects by suppressing mitochondrial reactive oxygen species (ROS) production and adenosine triphosphate biosynthesis after ischemic stroke.^1^, ^2^ When oxidative stress occurs after ischemic stroke, ROS react with the macromolecular substances, such as cellular proteins, nucleic acids, and lipids, leading to cell injury and death.^3^, ^4^, ^5^, ^6^ It has been proven that ROS could be scavenged by antioxidant enzymes, which are downstream molecules of nuclear factor erythroid 2‐related factor 2 (Nrf‐2), including superoxide (SOD), catalase (CAT), glutathione peroxidase (GSH‐Px), and glutathione (GSH).^7^

Nuclear factor erythroid 2‐related factor 2, a classical cytoprotective transcription factor, has been reported to impart protective effects against oxidative injury.^8^, ^9^ Nrf‐2 is combined with its inhibitor Keap‐1 as a heterodimer in the cytoplasm under normal conditions.^10^, ^11^ However, with hypoxia, Nrf‐2 moves into the nucleus after separating from Keap‐1, and its interaction with antioxidant response elements induces the expression of different antioxidant enzymes, such as SOD, CAT, and GSH, during ischemic stroke.^10^, ^12^, ^13^ Therefore, Nrf‐2 is considered a crucial factor in brain protection as it improves oxidation stress during stroke and may be an important molecular target with good potential application in neuroscience.^14^, ^15^ Nrf‐2 has been proven to be beneficial in the angiogenic potential of brain tissues by improving vascular endothelial growth factor (VEGF).^16^ VEGF has been shown to exert three distinct roles: neuroprotective effects throughout the acute phase, neurogenesis, as well as angiogenesis during the chronic stage following ischemic stroke.^17^ The present study assessed changes in VEGF expression during the acute phase of ischemic stroke and examined the underlying mechanism.

Based on above compelling evidences, we demonstrated the hypothesis that leonurine was associated with the Nrf‐2 pathway. Here, we initially described the impact of leonurine on Nrf‐2 expression and showed that the protective role of leonurine in a mouse model for permanent middle cerebral artery occlusion (pMCAO) was correlated to the upregulation of Nrf‐2, induction of antioxidant stress, and VEGF upregulation.

## 2 METHODOLOGY

### 2.1 Animals

Male ICR mice were obtained from Vital River. ICR Nrf‐2^−/−^ and Nrf‐2^+/+^ mice were a kind gift from Professor Chunyan Li (Department of Neurology, The Second Hospital of Hebei Medical University, Shijiazhuang, Hebei, China). The animals were provided with food and water ad libitum and kept in controlled conditions (12 hours/12 hours light/dark cycle, and temperature of 22 ± 3°C). All animal management and experiments were performed in accordance with the guidelines of the National Institutes of Health Guide for the Care and Use of Laboratory Animals.

### 2.2 Model of pMCAO

This study employed a classical stroke model as well as established the pMCAO model as described elsewhere.^18^, ^19^ The body weight of mouse, which was used during experimentation, was within the range of 27‐30 g. The animals were sedated with 10% chloral hydrate (400 mg/kg; Yongda Chemical Reagent Co., Ltd.) via intraperitoneal injection. A midline incision was created on the neck, followed by exposure of the right common carotid artery, external carotid artery, and the internal carotid artery. A preparation line (Beijing Shadong BioTechnoiogiies Co., Ltd.) was then inserted into the encephalic region, starting from the right external carotid artery, and then passing through intracranial segment of the right internal carotid artery. The line length was 10 mm apart from the common carotid artery bifurcation; the plug line was placed in the middle cerebral artery openings accordingly. The nylon suture was cut, followed by tightening artery nub, and suturing the skin and subcutaneous tissue.

### 2.3 Drug administration and experimental groups

Drug dose screening was initially performed. Male ICR mice received three doses of leonurine (5.0, 10.0, and 15.0 mg/kg in 0.9% normal saline: purity >99%; measured using HPLC; Xinxiang Tianfeng Fine Chemicals Co., Ltd.) approximately 2 hours after establishing the pMCAO model. Based on the results of neurological testing and the infarct volume of the mice 24 hours after pMCAO establishment, leonurine at a dosage of 10.0 mg/kg was determined to be the effective dose for therapy and utilized in the subsequent experiments. The animals were arbitrarily distributed into three groups, namely the Sham group that was given an equal volume of 0.9% normal sodium, the Vehicle group that was operated pMCAO and received an equal volume of 0.9% normal sodium, and the LEO10 group that received leonurine at a dose 10.0 mg/kg approximately 2 hours after pMCAO establishment by intraperitoneal injection. To determine the underlying mechanism by which leonurine influences brain tissues, the animals were then arbitrarily distributed into four groups, namely the Nrf‐2^+/+^ Vehicle group: male Nrf‐2^+/+^ mice were given an equal volume of 0.9% normal sodium around 2 hours after pMCAO; the Nrf‐2^+/+^ LEO10 group: male Nrf‐2^+/+^ mice were administered leonurine at a dosage of 10.0 mg/kg 2 hours after pMCAO via intraperitoneal injection; the Nrf‐2^−/−^ Vehicle group: male Nrf‐2^−/−^ mice were given an equal volume of 0.9% normal sodium approximately 2 hours after pMCAO; and the Nrf‐2^−/−^ LEO10 group: male Nrf‐2^−/−^ mice were administered leonurine at a dosage of 10.0 mg/kg 2 hours after pMCAO via intraperitoneal injection.

### 2.4 Neurological deficit scores

A neurological test was conducted 24 hours after pMCAO utilizing the modified scoring system of Longa et al, which used the followed scoring system: 0, no deficits; 1, difficulty in achieving a full extension of the contralateral forelimb; 2, inability to stretch out the contralateral forelimb; 3, mild circling toward the contralateral side; 4, severe degree of circling; and 5, falling toward the contralateral side. Higher neurological deficit scores are suggestive of a more severe disruption of motor function.

### 2.5 Brain infarct volume

Infarct volumes were quantified utilizing by 2,3,5‐triphenyltetrazolium chloride (TTC) approximately 24 hours after pMCAO. Brain tissues were processed to yield seven coronal sections (thickness: 1 mm), which were stained with 2% TTC, and then fixed with 4% paraformaldehyde. Images of the TTC‐stained sections were obtained and analyzed utilizing the ImageJ software to determine the infarct volume. To compensate for the occurrence of brain edema, hemisphere lesion volumes were quantified using the formula as previously reported^20^: the percentage hemisphere lesion volume (%HLV) = (Total infarct volume − [Volume of ipsilateral hemisphere‐Volume of contralateral hemisphere]/Contralateral hemisphere volume) × 100.

### 2.6 Western blotting

Proteins were extracted from the cerebral cortex of the right hemisphere utilizing a Total Protein Extraction Kit (Applygen Technologies Inc) and a nuclear‐cytosol extraction kit (Applygen Technologies Inc) 24 hours after establishment of pMCAO. Total protein for Nrf‐2, VEGF, and nuclear protein for Nrf‐2 were prepared. A BCA protein assay reagent kit was utilized to establish protein concentrations. Each protein sample (50 μg) was analyzed by sodium dodecyl sulfate polyacrylamide gel electrophoresis (SDS‐PAGE). The polyvinylidene difluoride (PVDF) membranes and the gel were together incubated at 4°C for 2 hours with steady current, and the proteins were transferred onto membranes. Then the membranes were blocked by incubating at room temperature for 1 hour with 5% skimmed milk. Various primary antibodies were hybridized onto the PVDF membrane by incubating overnight at 4°C. The primary antibodies employed in this study included rabbit anti‐Nrf‐2 (1:200, Santa Cruz Biotechnology) and rabbit anti‐VEGF (1:200, Santa Cruz Biotechnology). The following day, the membranes were rinsed with TPBS (PBS and 0.1% Tween‐20) thrice (10 minutes each wash). After that, fluorescent antibodies (goat anti‐rabbit or goat anti‐mouse, 1:10 000; Rockland) were added onto the membranes and incubated at 37°C for 1 hour. After incubation, the membranes were washed with TPBS thrice (10 minutes each wash). An imaging densitometer (LI‐COR Bioscience) was employed to determine the relative density of every band. H3 (1:200; Bioworld) and β‐actin (1:10 000; Santa Cruz Biotechnology) were used as reference. As previously reported, the Western blotting data of each group were, respectively, divided by the control group value to represent the relative protein levels.^21^

### 2.7 Real‐time polymerase chain reaction

Real‐time polymerase chain reaction (RT‐PCR) was conducted as described elsewhere.^15^ Nrf‐2 and VEGF mRNA levels were evaluated 24 hours after pMCAO. Total RNA extraction was performed using the cerebral cortex of the right brain hemisphere using TRIzol (Invitrogen) following the manufacturer's instructions. Reverse transcription was performed using a synthetic first‐chain cDNA toolkit (Fermentas International Inc) for quantitative PCR (MX 3005P) using a fluorescent dye (SYBR Green I; Cwbio). Absorbance of RNA was determined at wavelength of 260 and 280 nm with a UV spectrophotometer. The mRNA levels were normalized to that of β‐actin RNA. The forward and reverse primers were as follows: Nrf‐2, forward: GAAGCACGCTGAAGGCACAATG and reverse: GTTTGACACTTCCAGGGGCACTATC; VEGF, forward: ATCATGCGGATCAAACCTCACC and reverse: GGCTTTGTTCTGTCTTTCTTTGGTC; and β‐actin, forward: GCCTTCCTTCTTGGGTAT and reverse: GGCATAGAGGTCTTTACGG.

### 2.8 The activities of SOD, CAT, and GSH‐Px and the levels of MDA and GSH measurement

The cerebral cortex of the right hemisphere was collected 24 hours after pMCAO. The weight of the brain tissue was determined, then the tissues were immersed in normal saline nine times, and mechanical homogenization was performed in an ice box. The tissue homogenate at a concentration of 0.5%‐10% was then prepared and analyzed using SOD, CAT, GSH‐Px, MDA, and GSH kits following the manufacturers' instructions (A001, A007, A005, A003, and A006; Nanjing Jiancheng Bioengineering Institute, Nanjing, China). The activity of SOD was determined by xanthine oxidase assay, based on its reduction ability to superoxide anion radical. Ammonium molybdate could terminate CAT to decompose hydrogen peroxide, and the remaining hydrogen peroxide reacted with ammonium molybdate to produce yellow complex. We measured the change of yellow complex to explore the activity of CAT. The activity of GSH‐Px was represented by the speed of its catalyzing GSH. The levels of MDA were determined via measuring the red products, which were formed by the condensation of MDA with thiobarbituric acid. The levels of GSH were determined by measuring 5‐thio‐2‐nitrobenzoic acid, a kind of yellow substance, which was produced in the process of GSH oxidized by 5,5'‐dithiobis‐2‐nitrobenzoic acid.

### 2.9 ROS measurement

The cerebral cortex of the right hemisphere was collected 24 hours after pMCAO. Ophthalmic scissor was used to cut the brain tissue into pieces. Each sample was mixed with 1 ml of trypsin solution, and the suspension was incubated at 37°C. After 20 minutes, 3 ml of 0.01 M PBS solution was added to terminate digestion, and a nylon mesh was utilized to filter the brain tissue homogenate. The homogenate was centrifuged at 500 *g* for 10 minútes. Then, 1 ml of 0.01 M PBS solution was added to resuspend the cell pellet. ROS levels were assessed using a 2,7‐Dichlorodihydrofluorescein diacetate (DCFH‐DA) probe (E004; Nanjing Jiancheng Bioengineering Institute). ROS fluorescence intensities were measured using a microplate reader. The fluorescence intensities of each group were, respectively, divided by the control group to represent the relative ROS levels.

### 2.10 Nrf‐2^+/+^ and Nrf‐2^−/−^ genotyping of mice

The male and female mice were kept in separated cages at the age of four weeks. Each mouse was numbered individually, and the tip of tail (approximately 1 mm) was cut. DNA was then extracted for PCR analysis followed by 2% agarose gel electrophoresis at 100 V. After exposure to ultraviolet light, images of gel were obtained. A 400‐bp band indicated the Nrf‐2^−/−^ genotype, whereas a 750‐bp band reflected the Nrf‐2^+/+^ genotype.

### 2.11 Immunofluorescence staining

The mice were anesthetized at 24 hours after pMCAO for cardiac perfusion using 0.01 M PBS for 1 minutes, and rapid perfusion by 4% paraformaldehyde. The brains were dehydrated with 30% sucrose solution at 4°C for 48 hours, and then they were put into the −80°C refrigerator. Frozen 30‐μm thick sections were permeabilized using 0.5% Triton X‐100 for 10 minutes and blocked with 10% donkey serum for 1 hour at room temperature. Then, the brain sections were allowed to react overnight with primary antibodies at 4°C. The primary antibodies consisted of rabbit anti‐VEGF (1:50; Proteintech or 1:200; BOSTER), mouse anti‐GFAP (glial fibrillary acidic protein, 1:500; Abcam), rat anti‐CD31 (Cluster of Differentiation 31, 1:50; BD Biosciences), and mouse anti‐NeuN (Neuronal Nuclei, 1:500; Millipore Corporation). The tissue sections were hybridized with the corresponding secondary antibodies (Alexa Fluor 488 or 594, 1:800, Jackson Immuno Research) the following day at 37°C for 2 hours. Hoechst 33258 was utilized for identification of nuclei. Immunofluorescence images were obtained using an inverted fluorescence microscope, and the ischemic penumbra on the right hemisphere was chosen as the observation area.

### 2.12 Statistical analysis

Statistical analysis was performed using Statistical Product and Service Solutions (SPSS) 16.0, and data comparison among multiple groups was conducted with one‐way analysis of variance (ANOVA). When the variance was homogeneous and there were significant differences, s‐n‐k was further used for pairwise comparison, whereas a nonparametric rank sum test was used when the variance was not homogeneous. The neurological deficit score was compared using the Mann‐Whitney *U* test. Mean ± SEM (Standard Error of Mean) was used for all of the data except for the neurological deficit score. The test level was set to 0.05, and differences were deemed statistically significant at *P* < 0.05.

## 3 RESULTS

### 3.1 Leonurine decreased neurological deficit scores and volume of infarct in ICR mice after pMCAO

**Figure 1:**
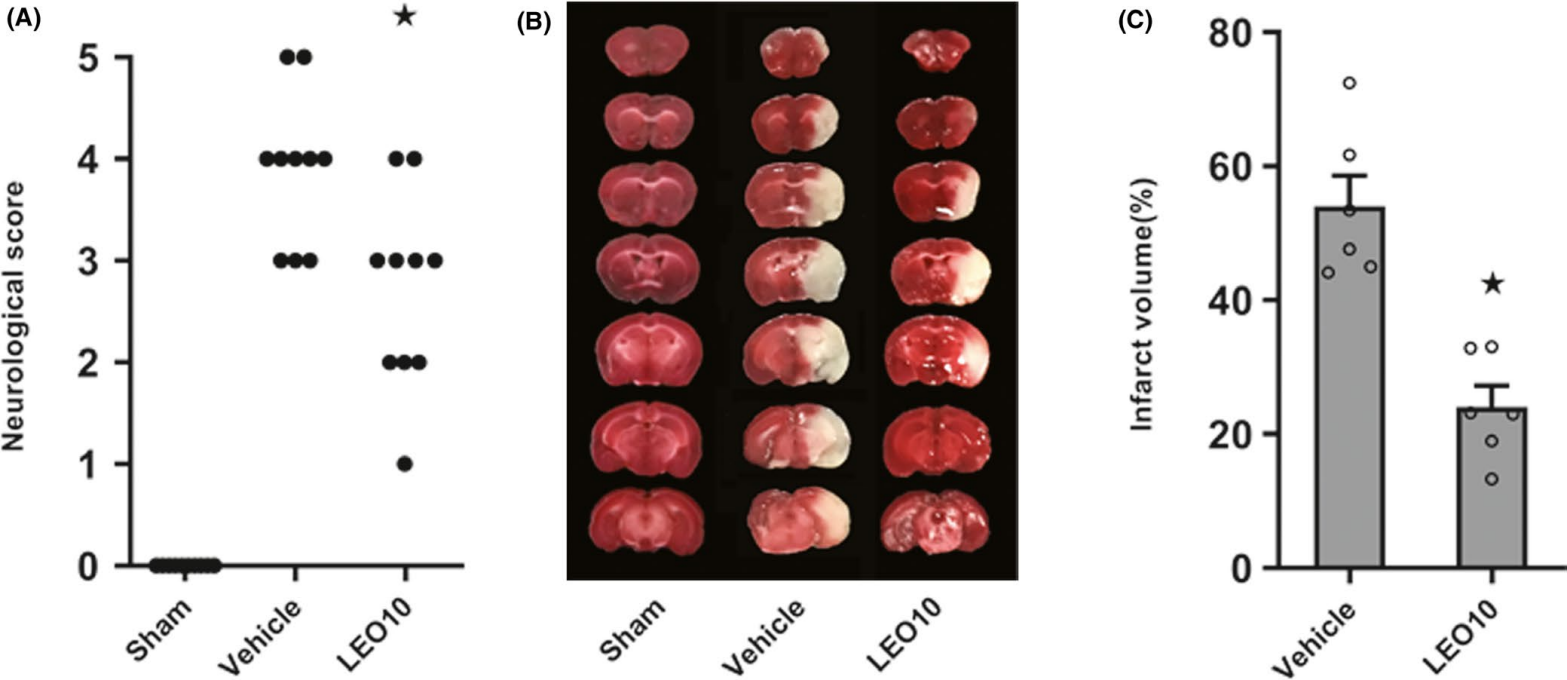
Leonurine decreased the neurological deficit values and infarct volume of ICR mice post‐pMCAO. The impact of leonurine on neurological deficit values (A), each circle indicates the score of each mouse. Compared to the Vehicle group, a significant improvement in neurological deficit scores was observed in the LEO10 group (*P* < 0.05) (n = 10 per group). Representative brain sections stained with TTC. Normal tissues showed deep red staining; meanwhile, the infarct portion shows pale gray staining (B). The impact of leonurine on the infarct volume relative to the Vehicle group significantly varied in the LEO10 group (*P* < 0.05) (C) (n = 6 per group). ^★^ *P* < 0.05 vs Vehicle group

To determine whether leonurine reduced ischemic stroke injury, measurement of neurological deficit scores and infarct volume was performed at 24 hours after pMCAO. Compared to the Vehicle group, a significant decrease in neurological deficit scores (*P* < 0.05; Figure 1A) as well as infarct volume (24.06% ± 3.17% vs 54.05% ± 4.54%, *P* < 0.05; Figure 1B,C) were observed in the LEO10 group.

### 3.2 Leonurine increased nuclear and total Nrf‐2 expression levels in ICR mice after pMCAO

**Figure 2:**
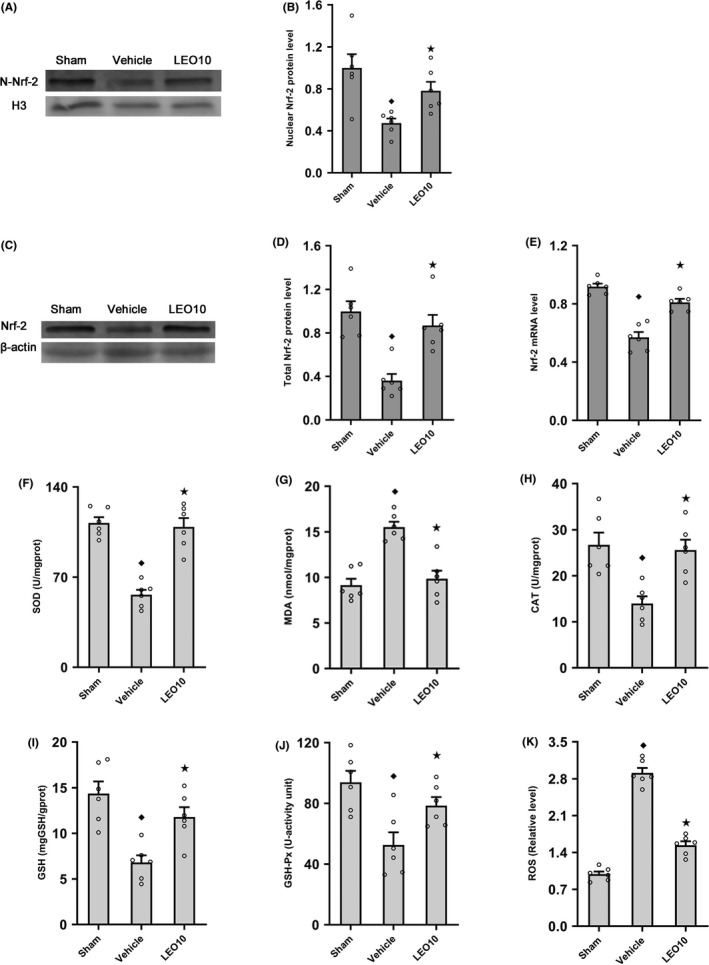
Leonurine influenced Nrf‐2 expression and regulated SOD, MDA, CAT, GSH, GSH‐Px, and ROS in ICR mice post‐pMCAO. Western blotting (A) nuclear Nrf‐2 protein expression among groups is shown. In contrast to the Vehicle group, nuclear Nrf‐2 protein expression (B) was upregulated in the LEO10 group post‐pMCAO (*P* < 0.05; n = 6 per group). The outcomes of Western blotting (C) examination of the expression of total Nrf‐2 among groups are shown. Compared to the Vehicle group, the expression of total Nrf‐2 protein (D) was upregulated in the LEO group (*P* < 0.05; n = 6 per group). RT‐PCR (E) examination of Nrf‐2 mRNA expression among groups is shown. In contrast to the Vehicle group, Nrf‐2 mRNA expression significantly rose in the LEO group (*P* < 0.05; n = 6 per group). In contrast to the Vehicle group, SOD (F), MDA (G), CAT (H), GSH (I), GSH‐Px (J), and ROS (K) significantly altered in the LEO10 group (*P* < 0.05; n = 6 per group). ^◆^ *P* < 0.05 vs Sham group. ^★^ *P* < 0.05 vs Vehicle group

To elucidate the mechanism underlying the protective effect of leonurine on brain tissues, we assessed nuclear Nrf‐2 and total Nrf‐2 protein expression by Western blotting, and Nrf‐2 mRNA expression levels by RT‐PCR in ICR mice 24 hours after pMCAO. Treatment with leonurine resulted in a significant increase in nuclear Nrf‐2 protein expression as compared to the Vehicle group after pMCAO (0.78 ± 0.08 vs 0.47 ± 0.04, *P* < 0.05; Figure 2A,B). Compared to the Vehicle group, total Nrf‐2 protein (0.87 ± 0.10 vs 0.36 ± 0.06, *P* < 0.05) and mRNA (0.81 ± 0.02 vs 0.57 ± 0.04, *P* < 0.05) expression levels were significantly increased in the LEO10 group (Figure 2C‐E).

### 3.3 Leonurine regulated SOD, MDA, CAT, GSH, and GSH‐Px and inhibited ROS production in ICR mice after pMCAO

To assess the antioxidant property of leonurine, the activities of SOD, CAT, and GSH‐Px, and the levels of MDA, GSH, and ROS 24 hours after pMCAO were assessed. Compared to the Vehicle group, SOD (108.93 ± 6.95 vs 56.30 ± 3.88, *P* < 0.05), CAT (25.59 ± 2.25 vs 13.99 ± 1.55, *P* < 0.05), GSH (11.78 ± 1.08 vs 6.81 ± 0.78, *P* < 0.05), and GSH‐Px (78.60 ± 5.53 vs 52.63 ± 8.35, *P* < 0.05) significantly increased, whereas that of MDA (9.85 ± 0.88 vs 15.51 ± 0.60, *P* < 0.05) significantly decreased in the LEO10 group (Figure 2F‐J). Compared to the Vehicle group, ROS (1.54 ± 0.07 vs 2.91 ± 0.10, *P* < 0.05) levels significantly decreased in the LEO10 group (Figure 2K).

### 3.4 Leonurine increased VEGF expression in ICR mice after pMCAO

**Figure 3:**
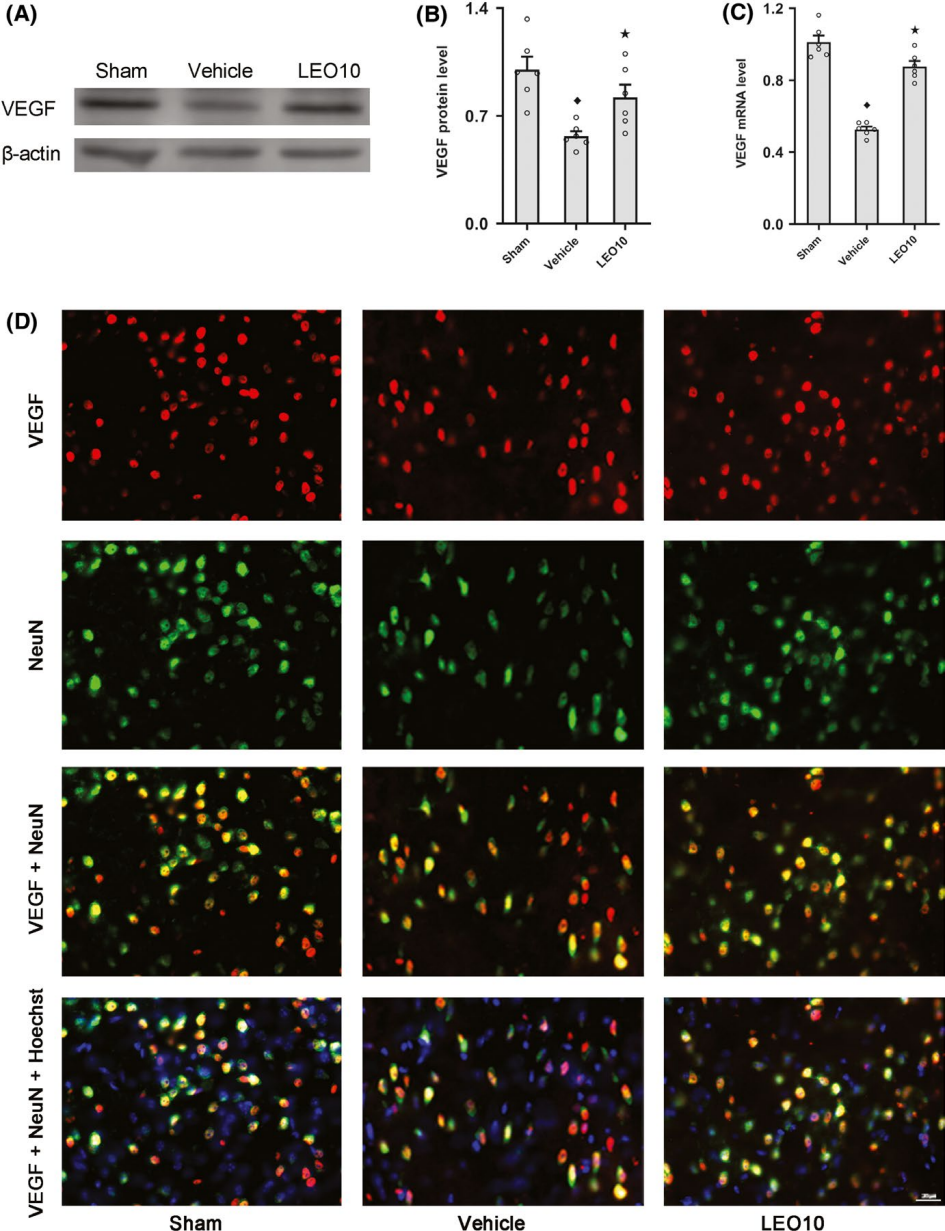
Leonurine influenced VEGF expression post‐pMCAO. In ICR mice, Western blotting (A, B) and RT‐PCR (C) analyses of VEGF among groups are shown. In contrast to the Vehicle group, VEGF expression was upregulated in the LEO group at protein and mRNA levels (*P* < 0.05; n = 6 per group). Immunofluorescence staining of VEGF in neurons of ICR mice after pMCAO, triple stained of brain sections using anti‐VEGF (red), anti‐NeuN (green), and Hoechst 33258 (blue) to indicate VEGF, neurons, and nuclei (D). VEGF was expressed in the majority of neurons and localized in the cytoplasm in Sham group. VEGF expression in neurons was decreased after pMCAO, which could be attenuated partly by leonurine. ^◆^ *P* < 0.05 vs Sham group. ^★^ *P* < 0.05 vs Vehicle group

To further investigate the mechanism underlying brain protection by leonurine, VEGF protein expression and mRNA expression levels were evaluated by Western blotting and RT‐PCR analysis 24 hours after pMCAO. Compared to the Vehicle group, VEGF protein (0.82 ± 0.08 vs 0.57 ± 0.03, *P* < 0.05) and mRNA (088 ± 0.03 vs 0.53 ± 0.02, *P* < 0.05) expression levels were significantly higher in the LEO10 group (Figure 3A‐C).

### 3.5 Localization of VEGF in the brain tissues of ICR mice after pMCAO

**Figure 4:**
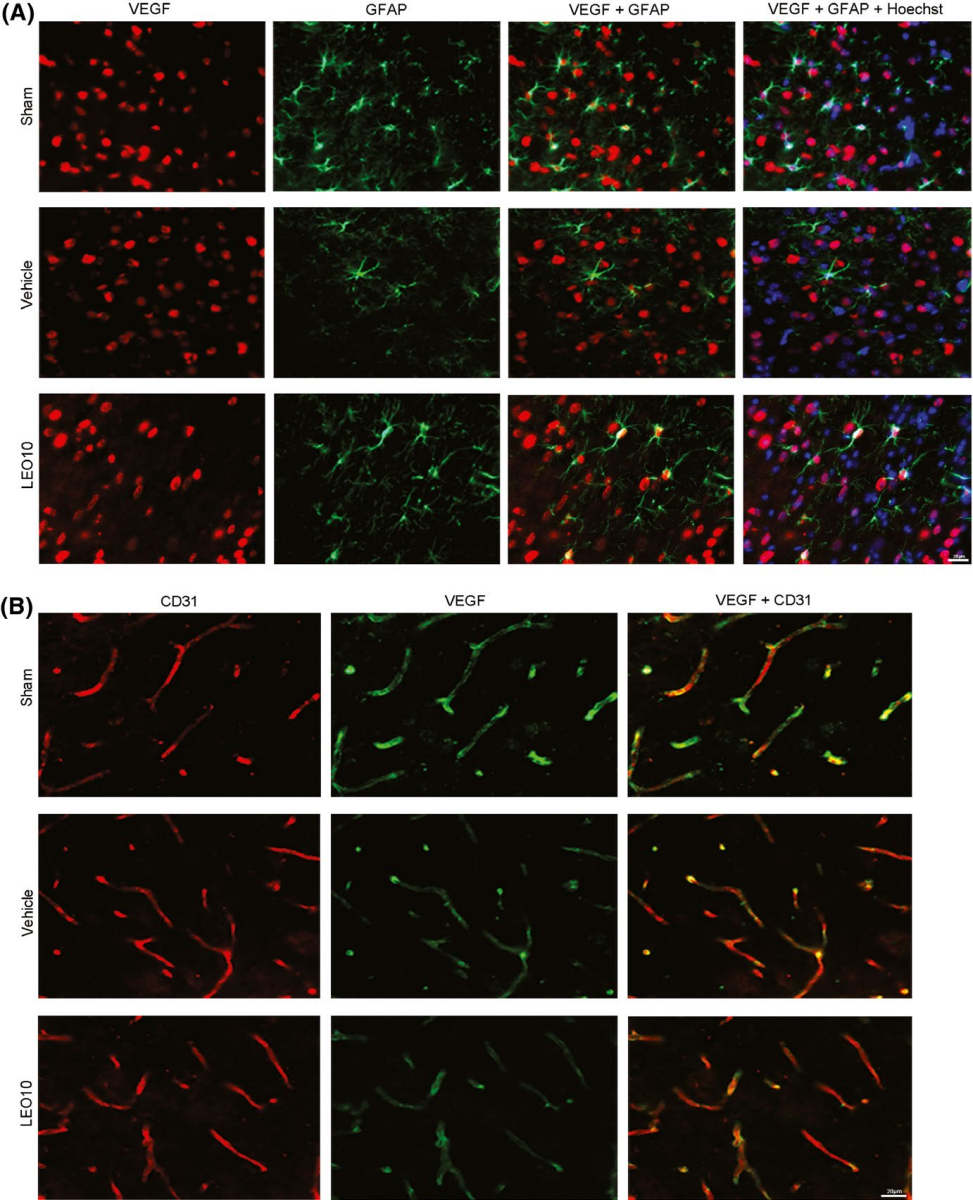
VEGF immunofluorescence staining of ICR mice astrocytes and vascular endothelium post‐pMCAO. Triple stained of brain sections using anti‐VEGF (red), anti‐GFAP (green), and Hoechst 33258 (blue) to indicate VEGF, astrocytes, and nuclei (A). VEGF was expressed in some astrocytes in the Sham group; VEGF expression in astrocytes was decreased after pMCAO. Nevertheless, the decrease in VEGF expression was alleviated by leonurine to some extent. Co‐staining of brain sections using anti‐CD31 (red) and anti‐VEGF (green) to respectively indicate vascular endothelium and VEGF (B), VEGF was expressed in some vascular endothelia in the Sham group. VEGF expression in vascular endothelium was reduced in the Vehicle group, but leonurine ameliorated the change after pMCAO

To localize VEGF, brain slices were stained with anti‐NeuN, anti‐GFAP, and anti‐CD31, respectively, to mark the neurons, astrocytes, and vascular endothelia in the brain tissues. VEGF was found to be expressed in a majority of neurons in the Sham group. VEGF expression was reduced in the neurons after pMCAO, which, however, could be partially prevented by leonurine (Figure 3D). VEGF was expressed in some astrocytes and vascular endothelia in the Sham group. VEGF expression decreased in the astrocytes and vascular endothelia after pMCAO, which were alleviated by leonurine to some degree (Figure 4A,D).

### 3.6 Leonurine did not impart neuroprotective effects on Nrf‐2^−/−^ mice after pMCAO

**Figure 5:**
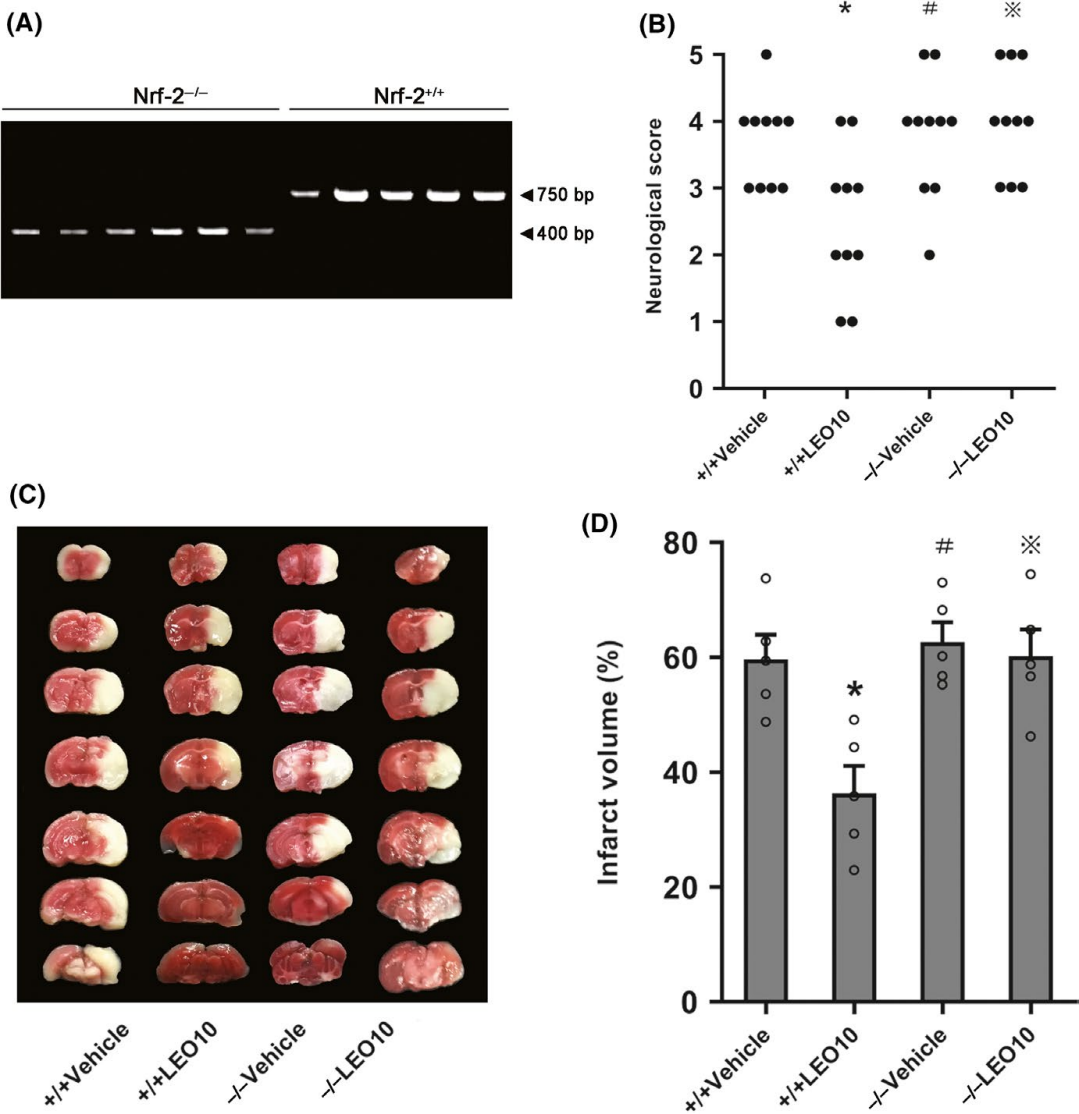
Leonurine did not exert neuroprotective effects on Nrf‐2^−/−^ mice after pMCAO. Genotyping (A) reveals that the bands of the Nrf‐2^−/−^ mice were 400 bp in size, and the bands of the Nrf‐2^+/+^ mice were at 750 bp in size. In Nrf‐2^−/−^ mice, the neurological deficit scores (B) (n = 10 per group) or infarct volume (C, D) (n = 5 per group) were not significantly ameliorated after leonurine treatment (*P* > 0.05). ^※^ *P* > 0.05 vs Nrf2^−/−^ Vehicle group. *P < 0.05 vs Nrf2^−/−^ LEO group. ^#^ *P* > 0.05 vs Nrf2^+/+^ Vehicle group

To determine whether the neuroprotective impact of leonurine occurred via Nrf‐2, the neurological deficit scores and volume of the infarct were evaluated in the Nrf‐2^−/−^ mice 24 hours post‐pMCAO. Compared to the Nrf‐2^−/−^ Vehicle group, neurological deficit scores (*P* > 0.05) or infarct volume (60.18% ± 4.66% vs 62.69% ± 3.42%, *P* > 0.05) did not significantly decrease in the Nrf‐2^−/−^ LEO10 group. Compared to the Nrf‐2^−/−^LEO10 group, neurological deficit scores (*P* < 0.05; Figure 5B) as well as infarct volume (36.31% ± 4.78% vs 60.18% ± 4.66%, *P* < 0.05; Figure 5C,D) were significantly reduced in the Nrf‐2^+/+^ LEO10 group.

### 3.7 Leonurine did not remarkably regulate SOD, MDA, CAT, GSH, GSH‐Px or inhibit ROS production in Nrf‐2^−/−^ mice after pMCAO

**Figure 6:**
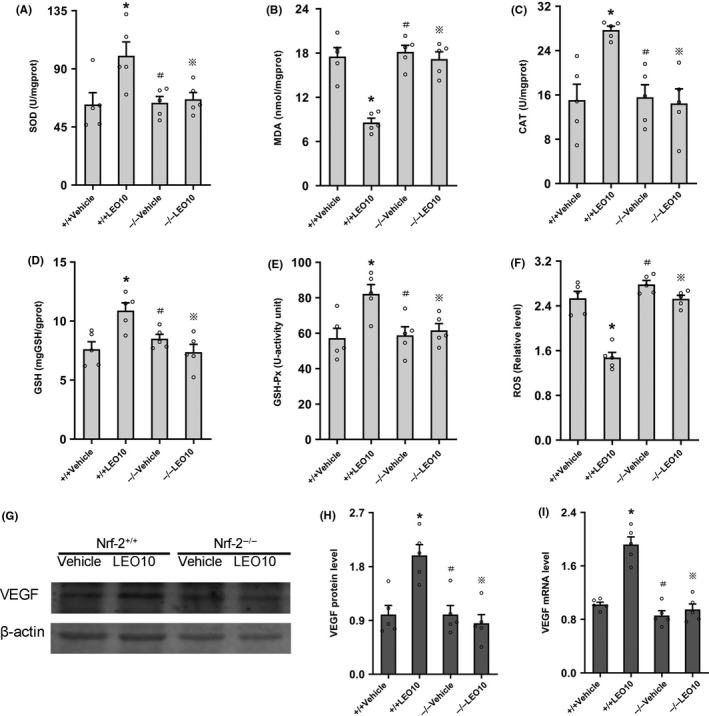
Leonurine did not remarkably regulate SOD, MDA, CAT, GSH, GSH‐Px, ROS, or VEGF in Nrf‐2^−/−^ mice after pMCAO. Leonurine did not remarkably regulate SOD (A), MDA (B), CAT (C), GSH (D), GSH‐Px (E), or inhibit ROS (F) production in Nrf‐2^−/−^ mice after pMCAO (*P* > 0.05; n = 5 per group). In Nrf‐2^−/−^ mice, Western blotting (G, H) and RT‐PCR (I) analysis of VEGF in different groups are shown. Compared to the Nrf‐2^−/−^Vehicle group, VEGF protein (*P* > 0.05) or mRNA (*P* > 0.05) expression was not significantly increased in the Nrf‐2^−/−^ LEO10 group. Compared to the Nrf‐2^−/−^ LEO10 group, VEGF protein (*P* < 0.05) and mRNA (*P* < 0.05) expression were upregulated in the Nrf‐2^+/+^LEO10 group (n = 5 per group). ^※^ *P* > 0.05 vs Nrf2^−/−^ Vehicle group. *P < 0.05 vs Nrf2^−/−^ LEO group. ^#^ *P* > 0.05 vs Nrf2^+/+^ Vehicle group

To elucidate the antioxidant mechanism of leonurine, we measured the activities of SOD, CAT, and GSH‐Px, and the levels of MDA, GSH, and ROS of the Nrf‐2^−/−^ mice 24 hours post‐pMCAO. Compared to the Nrf‐2^−/−^ Vehicle group, SOD (66.50 ± 5.21 vs 63.77 ± 4.86, *P* > 0.05), CAT (14.46 ± 2.61 vs 15.59 ± 2.24, *P* > 0.05), GSH (7.37 ± 0.66 vs 8.50 ± 0.38, *P* > 0.05), GSH‐Px (61.66 ± 3.73 vs 58.86 ± 4.82, *P* > 0.05), MDA (17.20 ± 0.97 vs 18.16 ± 0.89, *P* > 0.05), or ROS (2.53 ± 0.06 vs 2.78 ± 0.07, *P* > 0.05) did not significantly change in the Nrf‐2^−/−^ LEO10 group (Figure 6A‐F).

### 3.8 Leonurine did not markedly increase VEGF expression in Nrf‐2^−/−^ mice after pMCAO

To determine the underlying mechanism by which leonurine increased VEGF expression, we investigated VEGF expression in the Nrf‐2^−/−^ mice 24 hours post‐pMCAO. Compared to the Nrf‐2^−/−^ Vehicle group, expression of the VEGF protein (0.85 ± 0.15 vs 1.00 ± 0.15, *P* > 0.05) or mRNA levels (0.95 ± 0.08 vs 0.86 ± 0.07, *P* > 0.05) did not significantly increase in the Nrf‐2^−/−^ LEO10 group. Compared to the Nrf‐2^−/−^ LEO10 group, expression of the VEGF protein (1.99 ± 0.18 vs 0.85 ± 0.15, *P* < 0.05) and mRNA (1.92 ± 0.11 vs 0.95 ± 0.08, *P* < 0.05) was significantly higher in the Nrf‐2^+/+^ LEO10 group (Figure 6G‐I).

## 4 DISCUSSION

One major finding of the present study is that leonurine decreases neurological deficit scores as well as infarct volume postischemic stroke, triggers antioxidant responses, and increases VEGF expression by activating the Nrf‐2 pathway.

Leonurine, a traditional Chinese medicine, is widely used in clinical gynecology.^22^ Leonurine has been shown to impart antiapoptosis effects in ischemic stroke mice by upregulating B‐cell lymphoma/leukemia‐2 (Bcl‐2) and downregulating Bcl‐2‐associated X protein (Bax) expression.^1^ Leonurine has also been reported to exert antidepressant effects by improving monoamine neurotransmitters in depression model of mice.^23^ Leonurine plays an antineuroinflammatory role by inhibiting microglial overactivation in Alzheimer's disease Sprague Dawley (SD) rats.^24^ These findings suggest that leonurine has multiple biological effects on nervous system diseases and thus may be potentially used in therapeutic regimens.^25^, ^26^, ^27^, ^28^ In this evaluation, based on neurological deficit scores and infarct volumes, we determined that leonurine imparted neuroprotective effects during the early stages after ischemic stroke. Thus, we proposed that leonurine could be considered as a potential alternative drug for ischemic stroke therapeutics, and further elucidated the underlying mechanism of leonurine.

There is growing evidence suggesting that oxidative stress, which is a type of secondary brain injuries that occurs after a stroke, should be regulated in order for a patient to recover.^4^, ^5^, ^29^ A previous study has revealed that the Nrf‐2 pathway and their corresponding target genes impart neuroprotective effects against oxidative, excitotoxic, and metabolic damages, which aggravated ischemia, in both cell culture and animal models.^30^ In our present experiment, Nrf‐2 protein expression was decreased in MCAO mice as compared with sham mice. Previous in vivo and vitro studies have shown that Nrf‐2 protein expression decreased after oxidative damage.^9^, ^31^, ^32^ Our findings are consistent with previous researches, but some studies have suggested that Nrf‐2 had transported into the nucleus after ischemia and its expression increased.^15^, ^33^ These discrepancies in mechanism may be due to the use of different animal species and models of ischemic stroke, as well as variations in sampling time points and parts. According to our data from Western blotting, leonurine increased Nrf‐2 expression in the nucleus and the total Nrf‐2 protein expression after pMCAO. These results implied that leonurine increased the nuclear transposition of Nrf‐2 and might play an antioxidant role. Meantime, we are also aware that the reasons why leonurine promoted Nrf‐2 nuclear transposition should be further studied. RT‐PCR analysis revealed that leonurine increased Nrf‐2 mRNA levels and implied that leonurine upregulated Nrf‐2 expression at the level of transcription at least. Nevertheless, whether leonurine is related to the degradation of Nrf‐2, and whether leonurine has effect on Keap‐1 expression should be further examined.

It has been reported that ROS are mediators of the mitochondria, DNA repair enzymes, and transcription factors, which contribute to neuronal apoptosis.^34^, ^35^, ^36^ They are involved in the release of cytotoxic substances during oxidative stress responses after ischemic stroke.^37^ SOD has been reported to promote ROS to turn into harmless water molecules and oxygen molecules, and eliminate the lipid per‐oxidation of cells.^38^ Thus, antioxidant therapy should be concerned after stroke,^39^ and the removal of ROS or increasing the production of free radical scavengers may be used as targets for ischemic stroke treatment. It has been proved that activating Nrf‐2 promotes antioxidant responses by increasing SOD expression to protect brain tissue from ischemia after pMCAO.^14^ In the present study, according our data of assay kits, leonurine regulated the SOD, CAT, GSH‐Px, MDA, GSH, and inhibited ROS production after pMCAO. Our results suggested that leonurine played a neuroprotective role through promoting antioxidant stress.

Vascular endothelial growth factor exerts neuroprotective impacts during the early stages of ischemic stroke.^40^, ^41^ The application of VEGF to rats via intracerebroventricular injection 24‐72 hours post‐MCAO resulted in a reduction in infarct size during the acute phase, as well as the cerebral cortical region. VEGF also improved the general neuronal morphology and reduced the number of small and abnormally shaped neuron.^17^, ^42^ In recent studies, the damaged neurons may release VEGF, which contributes to promoting microglia/macrophage polarization and obtaining the potentially beneficial phenotypes in early stages of ischemic stroke.^40^ On the basis of our data from Western blotting and RT‐PCR in ICR mice, leonurine upregulated VEGF at the levels of both protein and mRNA. VEGF is widely expressed in the brain tissue, mainly through choroid plexus, neurons, astrocytes, and vascular endothelium.^43^, ^44^ However, investigations on VEGF localization during the acute phase of ischemic stroke are limited. Based on the immunofluorescence results, VEGF was expressed in the neurons, astrocytes, and vascular endothelium in brain tissues, which was in line with the findings of earlier studies. After the application of leonurine, VEGF expression was increased in neurons, astrocytes, and vascular endothelium. However, additional studies are needed to validate the influence of leonurine on VEGF expression. These results indicated that upregulation of VEGF, as well as antioxidant stress, might be responsible for neuroprotective effect of leonurine after ischemic stroke.

Then, we verified whether the neuroprotective effect of leonurine was achieved through Nrf‐2. According our data, leonurine had no noteworthy neuroprotective or antioxidant stress effect in Nrf‐2^−/−^ mice after pMCAO. The results of this study suggested that leonurine activated the Nrf‐2 pathway as a neuroprotective mechanism against the antioxidant stress.

Next, we further investigated why leonurine increased VEGF by observing the VEGF expression in Nrf‐2^+/+^ and Nrf‐2^−/−^ mice after ischemic stroke. In our Western blotting or RT‐PCR analysis, there was no notable improvement in VEGF expression after leonurine administration in the Nrf‐2^−/−^ mice. Then by comparing Nrf‐2^−/−^ LEO10 group with Nrf‐2^+/+^ LEO10 group, we found that leonurine could up‐regulate VEGF expression in Nrf‐2^+/+^ mice. However, leonurine could not significantly increase the VEGF expression in Nrf‐2^−/−^ mice. The relationship between Nrf‐2 and VEGF expression has been reported.^45^ Studies have shown that astrocytic VEGF release was increased after Nrf‐2 activation,^46^ the upregulation of VEGF was markedly inhibited in the Nrf‐2 knockdown rat,^47^ and VEGF‐Nrf‐2 positive feedback loop was observed in brain microvascular endothelial cells.^48^ These evidences suggest that leonurine may be utilized in inducing VEGF expression by activating the Nrf‐2 pathway.

In conclusion, leonurine exerts neuroprotective effects after ischemic stroke, and it plays a role in antioxidant stress and upregulation of VEGF. The potential mechanisms of these effects may be achieved through acting on the Nrf‐2 pathway.

## CONFLICT OF INTEREST

The authors declare no conflict of interest.
